# Dual targeting of mitochondrial function and mTOR pathway as a therapeutic strategy for diffuse intrinsic pontine glioma

**DOI:** 10.18632/oncotarget.24045

**Published:** 2018-01-08

**Authors:** Maria Tsoli, Jie Liu, Laura Franshaw, Han Shen, Cecilia Cheng, MoonSun Jung, Swapna Joshi, Anahid Ehteda, Aaminah Khan, Angel Montero-Carcabosso, Pierre J. Dilda, Philip Hogg, David S. Ziegler

**Affiliations:** ^1^ Targeted Therapies Research Program, Children’s Cancer Institute, Lowy Cancer Research Centre, University of New South Wales, Sydney, New South Wales, Australia; ^2^ Experimental Therapeutics Program, Children’s Cancer Institute, Lowy Cancer Research Centre, University of New South Wales, Sydney, New South Wales, Australia; ^3^ Preclinical Therapeutics and Drug Delivery Research Program, Department of Oncology, Hospital Sant Joan de Déu, Barcelona, Spain; ^4^ Biophytis, UPMC, BC94, Paris, France; ^5^ ACRF Centenary Cancer Research Program, Centenary Institute, University of Sydney, Camperdown, New South Wales, Australia; ^6^ Kids Cancer Centre, Sydney’s Children Hospital, Randwick, New South Wales, Australia

**Keywords:** DIPG, paediatric brain tumour, mitochondria, mTOR, PDGFR

## Abstract

Diffuse Intrinsic Pontine Gliomas (DIPG) are the most devastating of all pediatric brain tumors. They mostly affect young children and, as there are no effective treatments, almost all patients with DIPG will die of their tumor within 12 months of diagnosis. A key feature of this devastating tumor is its intrinsic resistance to all clinically available therapies. It has been shown that glioma development is associated with metabolic reprogramming, redox state disruption and resistance to apoptotic pathways. The mitochondrion is an attractive target as a key organelle that facilitates these critical processes. PENAO is a novel anti-cancer compound that targets mitochondrial function by inhibiting adenine nucleotide translocase (ANT). Here we found that DIPG neurosphere cultures express high levels of ANT2 protein and are sensitive to the mitochondrial inhibitor PENAO through oxidative stress, while its apoptotic effects were found to be further enhanced upon co-treatment with mTOR inhibitor temsirolimus. This combination therapy was found to act through inhibition of PI3K/AKT/mTOR pathway, HSP90 and activation of AMPK. *In vivo* experiments employing an orthotopic model of DIPG showed a marginal anti-tumour effect likely due to poor penetration of the inhibitors into the brain. Further testing of this anti-DIPG strategy with compounds that penetrate the BBB is warranted.

## INTRODUCTION

High grade gliomas remain one of the most intractable of all cancers [1]. DIPGs are the most common form of high-grade glioma to affect children and represent the most aggressive of all childhood malignancies. They are not amenable to surgical resection, radiotherapy has palliative value only and chemotherapy has been found to be ineffective [2, 3]. Recent whole genome sequencing studies have identified mutations in histone genes H3K27M encoding for H3.1 and H3.3 isoforms, as well as in *TP53* and *ACVR1*. Furthermore, up to 36% of DIPG cases exhibit gene amplifications of receptor tyrosine kinases (RTKs) such as platelet-derived growth factor receptor (PDGFR), where as mutations of the phosphatidylinositol-4,5-bisphosphate 3-kinase (PI3K) cascade and loss of phosphatase and tensin homolog play a potential role on the oncogenesis of DIPG [4–6]. While genetic changes can lead to the activation of RTKs and PI3K/AKT/mTOR pathway, other mechanisms may also contribute to their activation such as accumulation of reactive oxygen species (ROS) from mitochondria [7, 8]. Cellular redox impairment has been closely linked with the initiation and progression of cancer, especially if mutations can occur in critical genes like oncogenes or tumour suppressors, and resistance to chemotherapeutic agents [9, 10]. Interestingly, in DIPG significant differences were observed in gene expression profiles between H3.3K27M and H3.1K27M subtypes for oxidative stress and mitochondrial dysfunction pathways, while proteomic analysis of cerebrospinal fluid (CSF) and DIPG tumours have also highlighted elevated response to oxidative stress [11, 12].

Depending on its severity and duration, mitochondrial oxidative stress can have different biological effects on cancer cells and may promote apoptosis [13]. Targeting mitochondria have attracted a lot of interest, and multiple agents including natural compounds or chemotherapeutic agents such as arsenic-based therapies have been developed to selectively promote cytotoxic production of ROS in tumour cells [9, 13, 14]. Arsenic-based drugs, such as arsenic trioxide have been used in diseases such as acute promyelocytic leukemias while new compounds have been developed with improved toxicity profile [15]. PENAO (4-(N-(S-penicillaminylacetyl)-amino)phenylarsonous acid) is an organoarsenical drug which targets mitochondrial protein adenine nucleotide translocase (ANT) through crosslinking of cysteine residues Cys160 and Cys257 [16]. It has shown pre-clinical activity in a variety of cancers such as ovarian, breast, and pancreatic cancer [16–20]. PENAO is currently being tested in a Phase 1 clinical study in adults with refractory solid tumors, including brain malignancies (U1111-1133-4715 and ACTRN12612000908831). ANT is known to control key mitochondrial functions such as the exchange of ATP/ADP across the mitochondrial membrane, which is important for oxidative phosphorylation. Furthermore it also controls the permeability of the inner-mitochondrial membrane. Opening of the permeability transition pore by inactivating ANT allows the equilibration of small solutes (<1.5 KDa) across the inner-membrane. This leads to uncoupling of oxidative phosphorylation, increase in superoxide levels, loss of trans-membrane potential, decrease in oxygen consumption and ultimately apoptosis [17]. There are four isoforms (ANT1-4) each with different distribution in the body. ANT2 isoform has been found to be up regulated in different cancers [21–23]. PENAO is the only ANT inhibitor in clinical development.

Considering the role of RTK/PI3K/mTOR pathway in gliomagenesis as well as the elevated expression profile of genes associated with mitochondrial dysfunction and oxidative stress in DIPG, we hypothesized that targeting mitochondria and RTK/PI3K/mTOR pathway may represent a rational therapeutic strategy against this aggressive pediatric brainstem glioma. In this study we show the cytotoxic efficacy of PENAO as a single agent in a panel of primary DIPG cells as well as its effects on mitochondrial function and RTK/PI3K/mTOR pathway. Furthermore we report on the synergistic effects of PENAO with mTOR inhibitor temsirolimus and the potential of this combination therapy to be translated into the clinic.

## RESULTS

### ANT2 is a therapeutic target in DIPG cells that can be inhibited with PENAO

**Figure 1:**
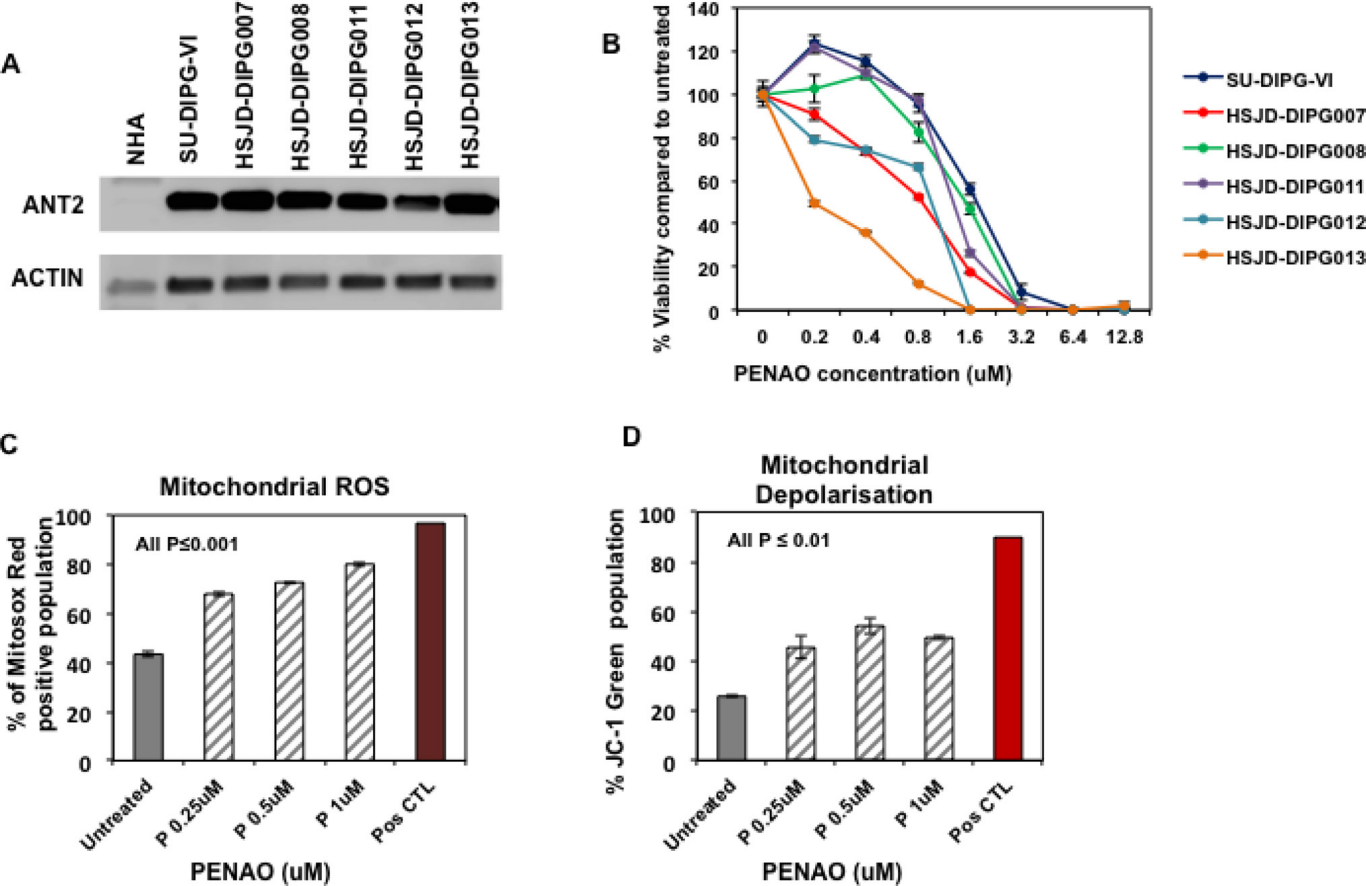
ANT2 is a potential therapeutic target for DIPG (**A**) Representative western blot image of ANT2 protein in a panel of DIPG cells and normal astrocytes (NHA); (**B**) Cytotoxic efficacy of PENAO tested for 72 h against a panel of neurosphere-forming DIPG cells. Viability was assessed by resasurin assay and presented as a percentage viability compared to untreated cells; Experiment was replicated 3 times; (**C**) Flow cytometric analysis of HSJD-DIPG007 cells for production of mitochondrial ROS. DIPG cells were treated with increasing concentration of PENAO (P) for 18 h and subsequently stained with MitosoxRed and analysed with FACS Canto B. Data represent average and SD of 3 determinations; untreated vs PENAO *p <* 0.001 for all concentrations tested; For positive control DIPG cells were treated with 100 uM antimycin for 1 h and analysed. (**D**) Flow cytometric analysis of HSJD-DIPG007 cells for mitochondrial depolarisation. DIPG cells were treated with increasing concentration of PENAO for 18 h and subsequently stained with JC1 and analysed with FACS Canto B. Data represent average and SD of 3 determinations; untreated vs PENAO *p <* 0.01 for all concentrations tested. For positive control DIPG cells were treated with 50 uM CCCP for 2 h and analysed.

ANT2 has been reported to be over-expressed in many cancer cells [21–23] therefore we evaluated its levels of expression by western blotting in primary DIPG cells. Compared with normal healthy astrocytes (NHAs), significantly higher protein levels were observed in a panel of six DIPG H3.3K27M mutated neurosphere cultures (Figure 1A). In view of this up-regulation we next sought to determine whether pharmacological inhibition of ANT2 with PENAO would affect the proliferation of DIPG cells grown as neurosphere-forming cultures. Using the resasurin-based cell viability assay, we found that PENAO exhibited cytotoxic activity against DIPG cells following 72 hours of treatment with inhibitory concentrations (IC_50s_) ranging from 0.2 to 1.5 µM (Figure 1B) where as a higher IC_50_ of approximately 8uM has been reported for NHA cells [17]. Although there is a small difference in the cytotoxic activity of PENAO among the panel of DIPGs it didn’t appear to correlate with ANT2 protein levels.

Since PENAO has been reported to influence mitochondrial function we then used flow cytometric analysis to determine whether PENAO could also affect mitochondrial integrity. Using Mitosox Red Dye we examined the production of mitochondrial ROS in HSJD-DIPG007 cells following treatment at various concentrations for 18h. In accordance with previously published studies in other cancer types [10, 24], we found that neurosphere-forming DIPG cells exhibit high baseline levels of ROS (Supplementary Figure 1). We found that PENAO was capable of inducing significant production of mitochondrial ROS in a dose-dependent manner (Figure 1C). Depolarization of the mitochondrial membrane is characteristic of loss of mitochondrial integrity and can be specifically monitored with fluorescent probe JC-1. Using JC-1 stain we therefore assessed mitochondrial depolarisation in PENAO-treated DIPG cells by measuring the decrease in red aggregates and subsequent shift in green fluorescence. We found that PENAO also significantly affects membrane potential following 18-hour treatments in a dose-dependent manner (Figure 1D). Taken together these data demonstrate that in DIPG cells a pharmacological intervention on ANT can lead to an inhibition of proliferation through an impairment of mitochondrial integrity.

### Pharmacological inhibition of ANT with PENAO influences PDGFR/PI3K/mTOR pathway and causes apoptosis

**Figure 2:**
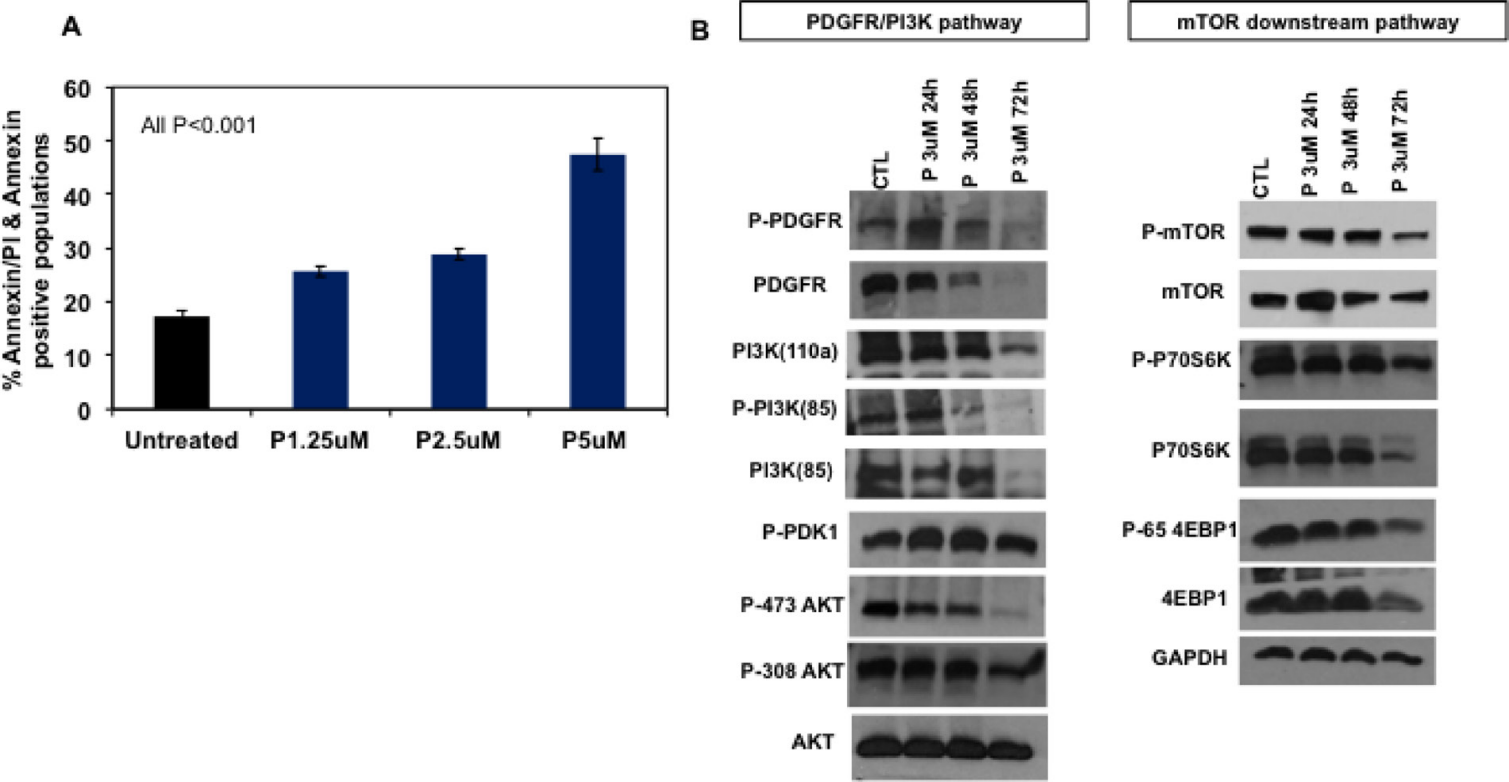
PENAO induces apoptotic cell death and affects major oncogenic pathways in DIPG cells (**A**) Flow cytometric analysis of HSJD-DIPG007 cells for apoptotic cell death. HSJD-DIPG007 cells were treated with increasing concentration of PENAO (P) for 42 h and subsequently stained with AnnexinV-FITC and 7AAD and analysed with FACS Canto B. Data represent average and SD of 3 determinations; untreated vs PENAO *p <* 0.01 for all concentrations tested. (**B**) Representative western blot images of key players involved in RTK/PI3K/mTOR pathway in HSJD-DIPG007 cells treated with PENAO as a function of time. DIPG cells were treated with 3 µM of PENAO for 24 h, 48, 72 h and subsequently lysed pellets were examined by western blotting.

Excessive mitochondrial damage and ROS production may be sufficient to overcome anti-apoptotic mechanisms activated in cancer cells and lead to induction of apoptosis. To assess the effect of PENAO on apoptosis in DIPG, HSJD-DIPG007 cells were treated with PENAO at increasing concentrations. 48 hours of treatment led to dose-dependent significant induction of apoptosis as measured by AnnexinV-FITC and propidium iodide staining (Figure 2A).

ANT2 knockdown with shRNA was previously found to inhibit receptor tyrosine kinase ErbB2 and PI3K in breast cancer cells [25]. Given that PDGFR is often overexpressed in DIPG, and the PI3K/mTOR pathway is one of the key oncogenic pathways implicated in the pathogenesis of DIPGs, we sought to examine the effect of ANT inhibition with PENAO on this pathway. Overall we found that treatment with PENAO led to potent inhibition of expression of multiple components of this pathway (Figure 2B, Supplementary Figure 2). Interestingly, PDGFRa phosphorylation status as well as total protein levels were significantly reduced by 48 hours of treatment performed in HSJD-DIPG007 and SU-DIPG-VI cells (Figure 2B, Supplementary Figure 2). Similarly, PDGFRa phosphorylation of PI3K-85 appeared lower with subsequent decreased total protein levels of PI3K subunits at a later time point. Although overall AKT levels were not significantly affected, in HSJD-DIPG007 cells PENAO treatment reduced phosphorylation of residue Ser473, which is regulated by mTORC2, while phosphorylation of Thr308, which is influenced directly by the PI3K pathway, was only marginally affected at a later time point. In contrast we did not observe any differences in AKT and P-AKT in SU-DIPG-VI cells. We next examined for effects downstream of the PI3K pathway and specifically on mTOR and its targets. We found that PENAO treatments influenced mTOR levels and phosphorylation by 72 hours and these effects were also reflected downstream on its direct targets P70S6K and 4EBP1 (Figure 2B) however in SU-DIPG-VI cells we only observed effects on 4EBP1 (Supplementary Figure 2). Overall these results indicate that PENAO can induce apoptosis in DIPG cells and inhibits PDGFRa with subsequent effects on the PI3K/mTOR pathway.

### mTOR represents a potential therapeutic target in DIPG and mTOR inhibition synergistically enhances the cytotoxic activity of PENAO and irradiation

**Figure 3:**
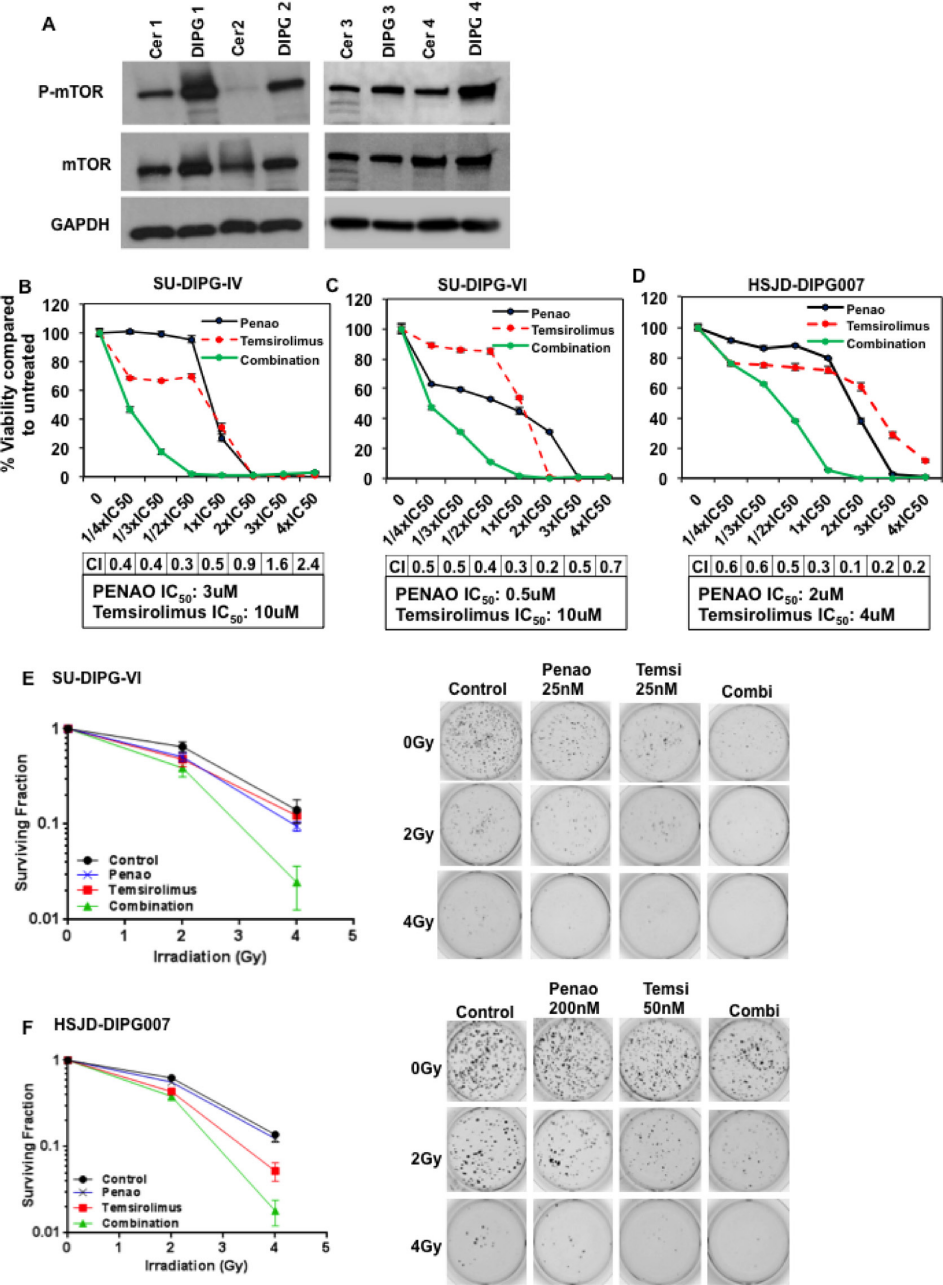
Inhibition of mTOR with temsirolimus enhances cytotoxic activity of PENAO and decreases clonogenic ability of DIPG cells (**A**) Representative western blot image of phosphorylated mTOR at Ser2448 as well as total mTOR levels in a panel of primary DIPGs and NHA cells (A), and patient DIPG autopsy samples with matched cerebellum (cer); (**B–D**) Cytotoxic efficacy of PENAO combined with temsirolimus tested at IC_50_ fractions in 3 neurosphere-forming DIPG cell lines. Viability was assessed by resasurin assay and presented as percentage viability compared to untreated cells; Synergy scores (CI) are included for each cell line. Experiment was replicated twice; each time *n* = 10. (**E–F**) Soft-agar colony assays performed in two DIPG cell lines show enhancement of irradiation upon combination with PENAO/temsirolimus; SU-DIPG-VI cells were treated with 25 nM of PENAO and 25 nM of temsirolimus, HSJD-DIPG007 cells were treated with 200 nM of PENAO and 50 nM of temsirolimus; Colonies were stained with MTT after 2 weeks and counted. Data represent surviving fractions; Experiment was performed twice *n =* 4 each time. SU-DIPG-VI, P/4Gy vs P/T/4Gy *p <* 0.01, T/4Gy vs P/T/4Gy *p <* 0.01; 4Gy vs P/T/4Gy *p <* 0.01; HSJD-DIPG007 P/4Gy vs P/T/4Gy *p <* 0.001, T/4Gy vs P/T/4Gy *p <* 0.05, 4Gy/P/T/4Gy *p <* 0.001.

Although mTOR has been suggested to play a fundamental role in gliomagenesis, it has not been previously reported to be upregulated in DIPGs compared to normal tissue. DIPG tissue from pediatric patient autopsies revealed higher phosphorylated mTOR levels compared with matched normal cerebellum (Figure 3A). Since PENAO influences mTOR and its downstream targets, we next sought to investigate whether the cytotoxic efficacy of PENAO could be enhanced with the mTORC1-specific inhibitor, temsirolimus. We found that temsirolimus dramatically enhanced the cytotoxic potential of PENAO on a variety of DIPG cells with combination indices indicating potent synergy at most of the concentrations tested (Figure 3B–3D). A similar synergistic effect was seen with other mTORC1 inhibitors such as rapamycin and everolimus (Supplementary Figure 3). Given that radiotherapy is the only current standard treatment available for DIPG patients, we therefore examined whether the combination of PENAO with temsirolimus could be enhanced with addition of irradiation. We performed soft-agar assays and found the clonogenic potential of DIPG cells was significantly impaired with the combination of temsirolimus and PENAO at nanomolar concentrations. This effect was further potently enhanced with the addition of irradiation in two primary DIPG neurosphere cultures (Figure 3E–3F). These data demonstrate that the clonogenic and proliferative nature of DIPG cells is severely impaired with combination of both agents, and that this combination can be further enhanced with irradiation.

### Combination of PENAO/temsirolimus inhibits further mitochondrial function and enhances apoptosis in DIPG neurospheres

**Figure 4:**
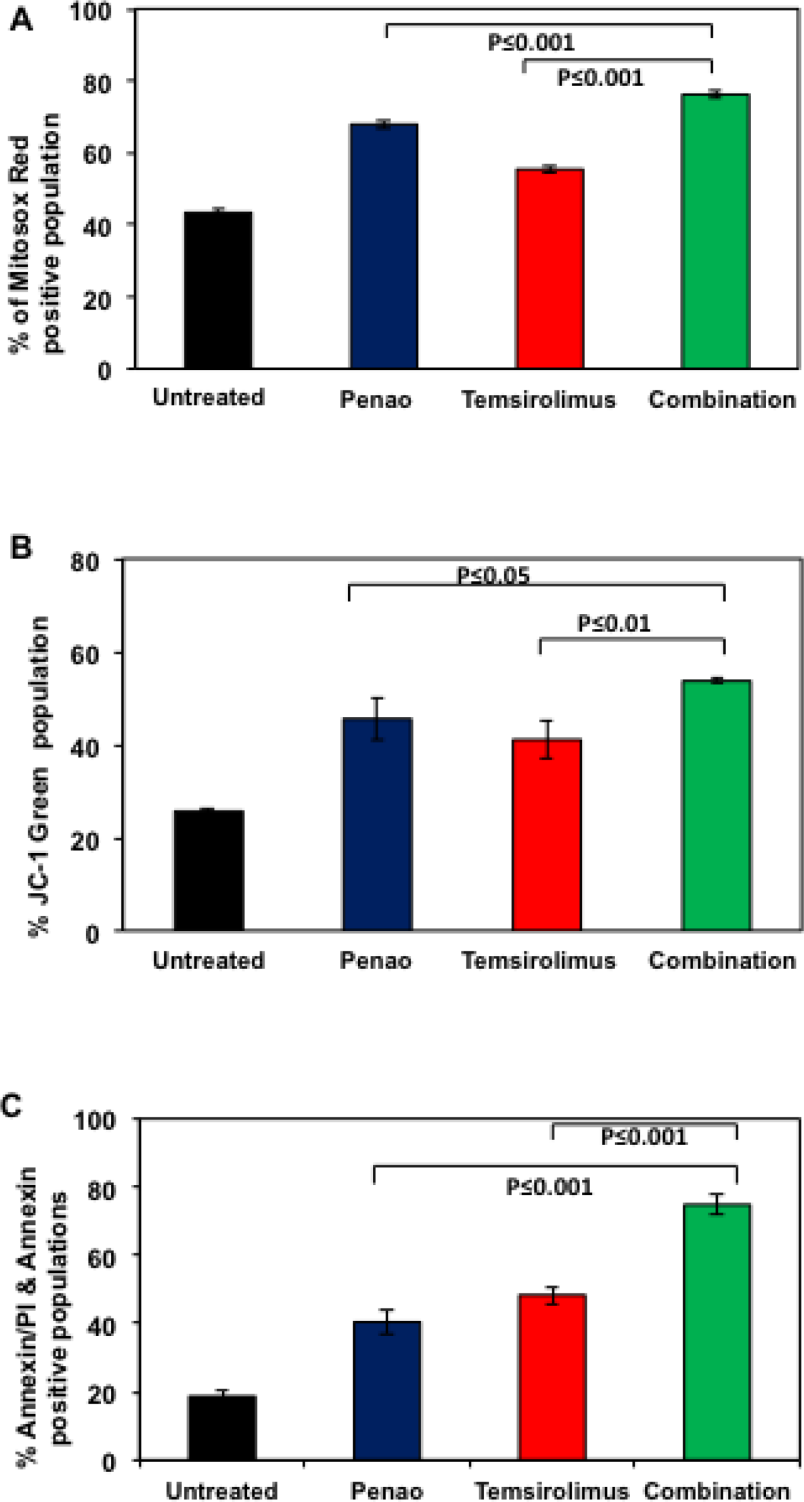
Combination of PENAO with temsirolimus increases mitochondrial dysfunction and enhances apoptosis (**A**) Flowcytometric analysis of HSJD-DIPG007 cells for production of mitochondrial ROS. DIPG cells were treated with 0.25 µM of PENAO, 2.5 µM temsirolimus, combined agents for 18 h and subsequently stained with MitosoxRed and analysed with FACS Canto B. Data represent average and SD of 3 determinations, PENAO vs Combination *p <* 0.001; temsirolimus vs Combination *p <* 0.001; (**B**) Flow cytometric analysis of HSJD-DIPG007 cells for mitochondrial depolarisation. DIPG cells were treated with 0.25 µM of PENAO, 2.5 µM temsirolimus, combined agents for 18 h and subsequently stained with JC1 and analysed with FACS Canto B. Data represent average and SD of 3 determinations; PENAO vs combination *p <* 0.05; temsirolimus vs combination *p <* 0.01; (**C**) Flow cytometric analysis of HSJD-DIPG007 cells for apoptotic cell death. DIPG cells were treated with 5 µM of PENAO, 10 µM temsirolimus, combined agents for 48 h and subsequently stained with AnnexinV-FITC and 7AAD and analysed with FACS Canto B. Data represent averages and SD of 3 determinations. PENAO vs combination *p <* 0.001; temsirolimus vs combination *p <* 0.001.

We next sought to test whether combination treatment with PENAO and temsirolimus further affects mitochondrial function and whether it enhances apoptotic cell death. Although mTOR inhibitors are known to promote autophagy and subsequent programed cell death, it has also been shown that mTOR inhibition could also affect mitochondrial function [26, 27]. We confirmed, using flow cytometric analysis, that temsirolimus promotes ROS production and membrane depolarisation (Figure 4A–4B). In addition, the combination of PENAO with temsirolimus led to a further significant increase in both parameters. As shown in Figure 4A mitochondrial ROS production increased from 67.5% and 55.4% for PENAO and Temsirolimus respectively as single agents, to 76.2% for the combination treated cells. Mitochondrial depolarisation also increased from 45.5% (PENAO) and 41% (Temsirolimus) to 53.7% for combination treated cells. We next examined whether the combination treatment could also enhance apoptotic cell death. Similar to effects seen on proliferation and clonogenic ability, the combination of PENAO and temsirolimus was also found to significantly enhance apoptotic cell death from 40.4% and 48% for PENAO and Temsirolimus respectively as single agents to 74.8% for combination treated cells (Figure 4C).

### Combination of PENAO and temsirolimus inhibits PI3K/AKT/mTOR signalling pathway with a decrease in ATP levels and activation of AMPK

**Figure 5:**
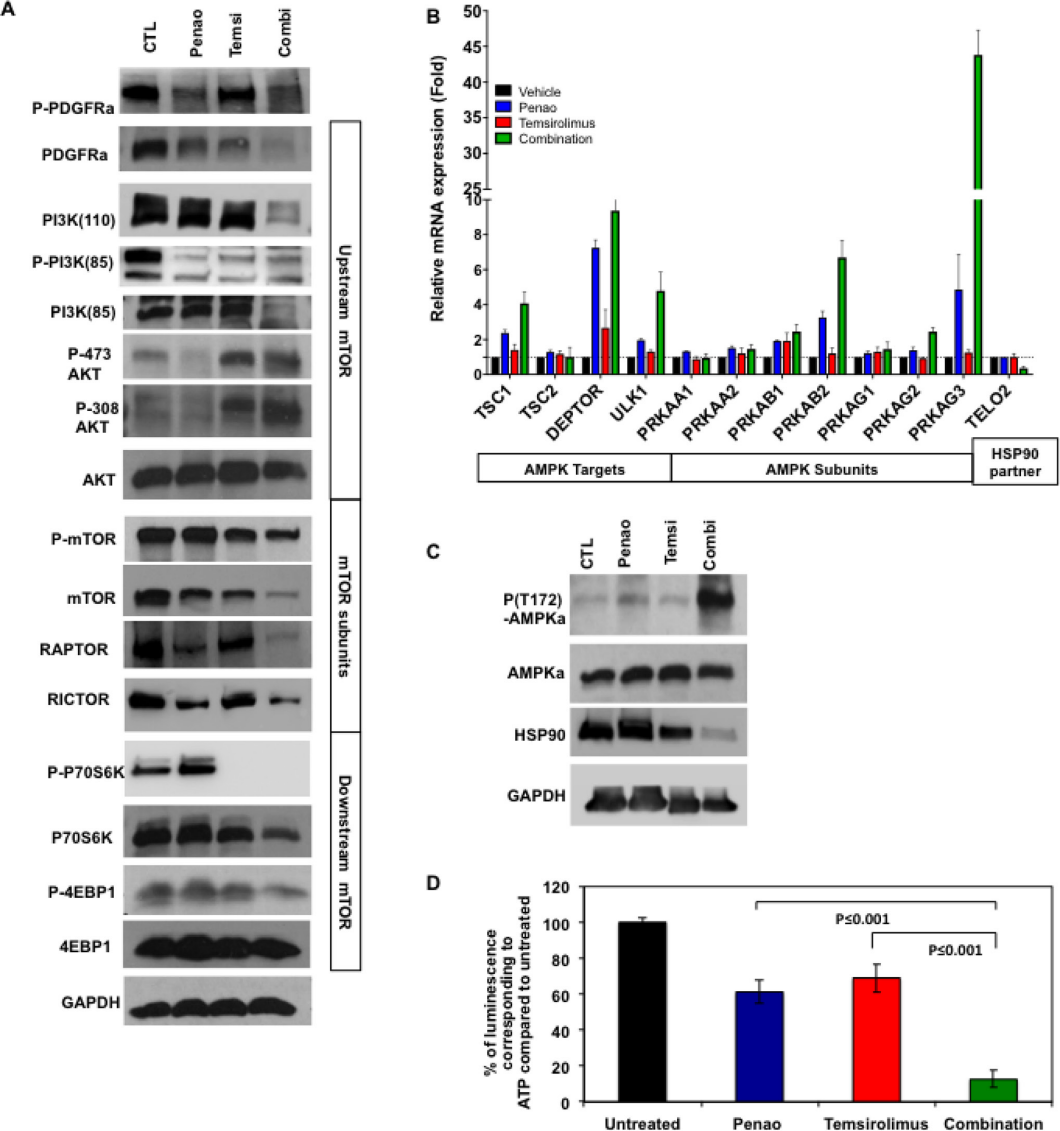
Combination of PENAO with temsirolimus affects PDGFRa/PI3K/mTOR pathway, HSP90 and activates AMPK (**A**) Representative image from western blot analysis of PDGFRa/PI3K/mTOR pathway in HSJD-DIPG007 cells treated with PENAO, temsirolimus and dual therapy for 48 h. Significant decrease is observed in protein levels in PDGFR, PI3K subunits, mTOR and its targets (**B**) Expression analysis of a panel of genes involved in AMPK pathway in HSJD-DIPG007 cells treated with PENAO, temsirolimus and dual therapy for 48h. Significant increase in mRNA levels of AMPK subunits and its targets *Tsc1*, and *Deptor* is observed, (*p* values for: *PRKAB1*: V vs C < 0.05, P vs C < 0.05, T vs C < 0.01, *PRKAB2*: V vs C < 0.01, P vs C < 0.05, T vs C< 0.05; *PRKAG1*, V vs C ns, P vs C ns, T vs C ns; *PRKAG2*, V vs C < 0.01, P vs C < 0.05, T vs C < 0.01; *PRKAG3*, V vs C < 0.05, P vs C < 0.05, T vs C < 0.05; *TSC1*, V vs C < 0.05, P vs C < 0.05, T vs C < 0.05; *DEPTOR*, V vs C < 0.01, P vs C < 0.05, T vs C < 0.001; *TEL*2, V vs C < 0.05, P vs C < 0.05, T vs C < 0.05); *ULK1* V vs C < 0.05, P vs C < 0.05, T vs C < 0.05; (**C**) Representative image from western blot analysis of mTOR regulators AMPK and HSP90 in HSJD-DIPG007 cells treated with PENAO, temsirolimus and dual therapy for 48 h. Significant decrease is observed in phosphorylation of AMPK indicating activation and protein levels of HSP90. (**D**) ATP determination in HSJD-DIPG007 cells treated with PENAO, temsirolimus and dual therapy for 48h. Significant decrease in ATP levels is observed in PENAO and combination treatments; Experiment performed twice each time *N =* 5; P vs C *p <* 0.001, T vs C *p <* 0.001.

Having established that PENAO and temsirolimus enhance cytotoxicity, apoptotic cell death and influence mitochondrial function, we next sought to assess whether the combination therapy acts on the PDGFR/PI3K/mTOR pathway at the protein level. Using immunoblot analysis, we found that the combination led to a global reduction in activity of the mTOR pathway both upstream and downstream of mTOR protein (Figure 5A). We observed lower levels of total PI3K and PDGFRa levels, whereas phosphorylation of both proteins appears to be reduced in single agent treatments rather than combination. In contrast total protein levels of PI3K and PDGFRa are seen reduced in the combination treatment. Furthermore we found decreased total and phosphorylated mTOR levels. We also observed reduced protein levels of 4EBP1 indicating that this combination therapy can affect downstream mTOR targets. However, similar to effects seen in other cancers [28, 29], we also observed an increase in phosphorylation of AKT at both S473 and T308 subunits, which is indicative of mTORC2 activation. This suggests both that AKT is activated as a rebound phenomenon, and that mTOR inhibition ensues despite this activation, suggesting that there is inhibition of mTOR that occurs independent of the PI3K pathway.

To examine this further, and since the combination of PENAO and temsirolimus treatment enhanced oxidative stress we then sought to determine whether mTOR inhibition is mediated by AMPK, which has been known to negatively regulate mTOR [30, 31]. Gene expression analysis performed with RT2 profiler array for mTOR regulatory pathway indicated an increase in genes that encode for AMPK subunits as well as its targets such as *Deptor* and *Tsc1*, which are known to directly inhibit mTOR function (Figure 5B). In addition we also observed an increase mRNA levels of autophagy marker *Ulk1* previously reported to be regulated by AMPK as well as mTOR [32]. Furthermore, at the protein level we found a significant increase in AMPK phosphorylation at residue T182 (Figure 5C), which has been implicated with AMPK activation during oxidative stress and low nutrient conditions such as low ATP levels. Since ANT plays an important role on ATP/ADP exchange we then sought to investigate the effect of each treatment on cellular ATP levels. As expected we found that PENAO treatments significantly lowered ATP levels, while combination with temsirolimus potently reduced these levels even further (Figure 5D). ATP levels are known to influence not only subsequent effects mediated by AMPK but also key players such as heat shock protein 90 (HSP90), which are responsible for protein stability [33]. In particular, HSP90 has been shown to enhance RTK as well as PI3K stability, which are dependent on ATP levels [34]. Disruption of HSP90 function due to low ATP levels has been found to direct oncogenic proteins such as RTKs towards a degradation fate [25, 35, 36]. We found that together with low intracellular levels of ATP there was also a significant decrease in HSP90 levels (Figure 5C). To further elucidate the role of HSP90 we performed proliferation assays and observed PENAO treatment could be enhanced significantly with addition of HSP90 inhibitor 17AAG, while the combination of both agents decreased significantly the protein levels of mTOR (Supplementary Figure 4). These results suggest that PENAO/temsirolimus treatment disrupts mitochondrial integrity and intracellular levels of ATP, and together influence the function of key positive and negative mTOR regulators PI3K, HSP90 and AMPK.

### *In vivo* combination treatment extends marginally the survival of a DIPG orthotopic model

**Figure 6:**
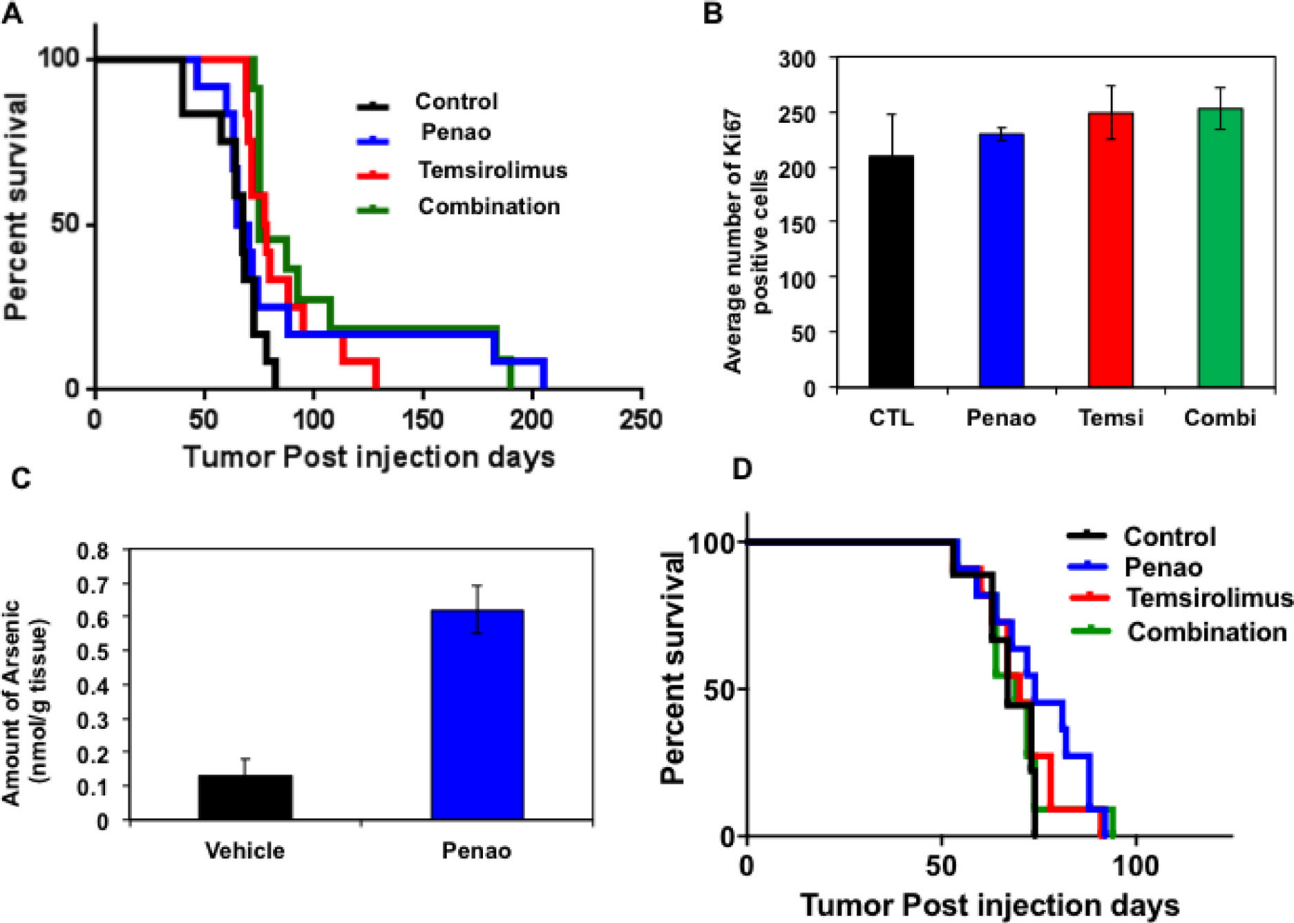
Therapeutic efficacy of PENAO, temsirolimus and combination in the HSJD-DIPG007 orthotopic animal model (**A**) Survival curve of orthotopicaly-injected DIPG animals treated with vehicle, PENAO administered by alzet Pump (3 mg/kg/day 7 days/week, 4 weeks), temsirolimus (10 mg/kg/day 5 days/week 2 weeks and 5 mg/kg/day 5 days/week 2 weeks) and combination. Each treatment cohort consisted of 12 animals and tumour engraftment was assessed by neurological symptoms and/or weight loss. At endpoint animals were sacrificed and brains were examine histologically with H&E and Ki67 stains. (**B**) Histological analysis of harvested brains from each treatment cohort. Ki67 positive cells were quantified in 6 separate fields of view in the pons region from 3–4 animals per cohort. (**C**) Mass spectrometric analysis of arsenic levels in brains from vehicle and PENAO-treated DIPG xenografts. (**D**) Survival curve of orthotopicaly-injected DIPG animals treated with vehicle, PENAO administered intraperitonealy (10 mg/kg/day 5 days/week), temsirolimus (10 mg/kg/day 5 days/ week) and combination for 4 weeks. Each treatment cohort consisted of 12 animals and tumor engraftment was assessed by neurological symptoms and/or weight loss.

We next sought to extend these findings to an *in vivo* system using a primary patient derived DIPG xenograft model. In order to mimic the clinical scenario we injected DIPG cells orthotopically in the 4th ventricle of immune-compromised mice. As we have previously described, this leads to infiltration of DIPG cells in pons, midbrain and cerebellum tumor, characterized by clinical signs of neurological decline such as ataxia, circling, head tilting as well as weight loss [37]. Four weeks after intracranial injection of DIPG cells, ALZET osmotic pumps were subcutaneously implanted and mice were treated with PENAO at 3 mg/kg/day for 2 weeks. Subsequently, the pumps were replaced and mice were treated for additional 2 weeks. Another cohort of mice received temsirolimus 10 mg/kg/day intraperitonealy (IP) for 2 weeks followed by another 2 weeks of treatment with 5 mg/kg/day. The control cohort was implanted with saline-containing pumps while the combination cohort received PENAO and temsirolimus treatments for a total period of 4 weeks. During the treatment period we did not observe any significant toxicity apart from some mild weight loss (10%), which was overcome with supplementation of wet food pellets at the bottom of the cages. Overall we observed that PENAO treatment, as a single agent did not significantly change median time to progression, although a few animals did have prolonged survival (Figure 6A). Compared with vehicle cohort, temsirolimus treatment only marginally enhanced the survival of mice whereas combined treatment with both agents did not significantly influence the overall lifespan. Similar to the PENAO-treated animals, combination treatment did not affect median survival but appeared to prolong the survival of a few animals. At endpoint, brains were harvested and histologically assessed for proliferation marker Ki67. We generally found that in all treatment cohorts DIPG cells infiltrated the brainstem and cerebellum regions in similar manner to vehicle treated mice. In addition, no significant changes were observed in the overall amount of proliferative Ki67-positive cells in brainstem and cerebellum regions (Figure 6B). We then investigated a higher dose of PENAO administered intraperitoneally (10 mg/kg/day 5days/week for 4 weeks) as single agent and in combination with temsirolimus (10 mg/kg/day 5 days/week for 4 weeks). Following this regimen we also didn’t observe any significant extension in the survival of the treated mice (Figure 6D). Since it has been suggested that drug failure in brainstem gliomas may be related to their intact blood brain barrier (BBB) we then evaluated the presence of arsenic atoms in DIPG xenografts treated with PENAO (Figure 6C). We found that the amount of arsenic atoms present were at the lowest level that could be detected by Inductively Coupled Mass Spectrometry (ICMS) suggesting that the lack of therapeutic efficacy in DIPG-engrafted mice is most likely due to limited drug delivery.

## DISCUSSION

DIPGs are highly proliferative tumors with a propensity to infiltrate and spread beyond the brainstem including cerebellum and midbrain. Due to their critical location and infiltrative nature, this tumor cannot be removed surgically. Irradiation is the only standard treatment, and although it offers temporary relief from the symptoms, almost all patients still succumb to the disease [3]. One of the key oncogenic pathways in DIPG with the potential to be therapeutically targeted is the PDGFR/PI3K/mTOR signaling network [3]. Interestingly, high expression levels of PDGFR have been reported in DIPG tumors independent of its genomic status, indicating that it could be therapeutically exploited in a wide population of DIPG patients [38]. Apart from genomic alterations, production of ROS has also been suggested to play a role in RTK activation such as for EGFR and PDGFR, either through increased activity in mitochondrial respiratory chain or cytosolic NADPH oxidases leading to subsequently aberrant downstream PI3K/AKT signaling [7]. In addition inactivation of PTEN through oxidation of cysteine residues has also been reported to mediate PI3K activity [39]. Although oxidative stress is responsible for mediating DNA damage and promoting oncogenic signaling, depending on the severity and duration it may influence cell survival or mediate apoptosis.

In this study we investigated the therapeutic potential of targeting mitochondrial ANT2, which has been demonstrated to play an important role in energy metabolism, mitochondrial integrity, oxidative stress and apoptosis. We found that DIPG primary cultures express high levels of ANT2 protein compared with nearly undetectable levels in NHAs, and that it serves as a potential therapeutic target for this aggressive paediatric disease. The over-expression of ANT2 in particular results in an anti-apoptotic role signal in cancer cells [17–19]. Pharmacological inhibition of this mitochondrial protein with PENAO led to low micromolar efficacy in a panel of primary neurosphere-forming DIPG cells. Furthermore, we observed an increase in mitochondrial ROS production as well as membrane depolarization, suggesting that PENAO can mediate its effects through mitochondrial function and subsequently induce apoptosis in DIPG cells. We also found that PENAO treatment leads to a significant decrease in PDGFR phosphorylation and protein levels, while subsequent effects on AKT and mTOR signaling appeared to be influenced at a later timepoint indicating that PENAO potentially mediates its initial effects through degradation of PDGFRa.

Since DIPG is one of the most chemoresistant pediatric tumors it is very likely that combination treatments will be needed to produce significant survival benefit in patients. mTOR has been implicated in gliomagenesis and is a key downstream player along the RTK/PI3K pathway [40, 41]. We found that mTOR is highly expressed in DIPG tumour tissue. Dual pharmacological inhibition of ANT and mTOR resulted in significant cytotoxic activity against neurosphere forming DIPG cells, secondary to mitochondrial dysfunction and apoptotic cell death. We subsequently found that combination treatment synergistically affected PDGFR/PI3K stability. In agreement with previously reported acquired resistance to rapalogues (such as rapamycin, temsirolimus), we observed increased phosphorylation of AKT at residues S473 and T308, which indicated activation of mTORC2 [42, 43]. Despite this, we observed a significant decrease in mTOR phosphorylation and overall protein levels indicating that this combination is capable of influencing mTOR pathway through alternative mechanisms. AMPK has been regarded as a key energy- and redox-sensing protein [44]. In fact, it has been suggested that inhibition of ANT can reduce the ATP/ADP ratio and subsequently activate AMPK through phosphorylation at Thr172 [45, 46]. In accordance with these reports, we found that PENAO treatment led to a significant decrease in ATP levels as well as an increase on AMPK phosphorylation. AMPK has been reported to inhibit mTORC1 complex through TSC1/2 as well as phosphorylation of mTOR partner protein Raptor [30, 31]. In addition, resveratrol-mediated AMPK activation has been suggested to exert its mTOR inhibitory function through increased Deptor protein levels [47]. We observed a significant increase in *Tsc1* and *Deptor* mRNA expression further suggesting that PENAO exerts and mTOR inhibitory effect via AMPK.

There are other important regulators of the RTK/PI3K pathway, such as HSP90. In cooperation with chaperone TEL2, HSP90 can mediate the activity, stability and formation of many protein complexes that act along the RTK/PI3K pathway, including mTORC1 [48–50]. Interestingly, we did observe a significant decrease in *Tel2* mRNA expression and HSP90 protein levels in our combination treatment and these results coincided with a decrease in PDGFRa, PI3K and mTOR. Specific cysteine residues in HSP90 have been found to play an important role for its chaperone activity [51]. Previous reports have indicated that arsenic-based compounds such as arsenite and GSAO can interact with these cysteine residues and interfere with HSP90 activity [51, 52]. Since PENAO is a GSAO analogue, it is possible that it is also directly inhibiting HSP90 thus affecting the stability and phosphorylation of PDGFRa, PI3K and mTORC1.

Despite the significant *in vitro* cytotoxic efficacy and reduced clonogenic ability of PENAO/temsirolimus combination with irradiation, this therapeutic approach did not extend significantly the survival in our DIPG orthotopic animal model. This is likely due to the low accumulation of PENAO secondary to an intact BBB. We found low nanomolar concentrations of PENAO in the brainstem region, which is below the IC_50_ range for the HSJD-DIPG007 xenograft. Furthermore, PENAO has been suggested to be exported by MRP1/2 transporters, which have been previously detected in DIPG, therefore lack of therapeutic efficacy is most likely due to inadequate delivery to the tumour [18, 53]. Perhaps the employment of direct routes of administration such as convection enhanced delivery (CED) may potentially improve therapeutic outcomes for this challenging disease [54]. Since there are currently very few readily available DIPG xenograft models, we have not tested this regimen in other models to date. It is possible that the activity may differ in other, although given that the concentrations of PENAO achieved in the brain were lower than the IC50 of all cultures tested, the probability of the treatment showing greater efficacy in other models seems low [37, 55–57].

Despite activation of the PI3K/AKT/mTOR pathway we did not observe enhanced survival upon treatment with temsirolimus. Inefficacy of temsirolimus has been mainly attributed due to upregulation of mTORC2 and although clinical trials have shown it to be well tolerated in pediatric patients it may be necessary for this drug to be explored therapeutically in combination with other therapies [58, 59]. Interestingly, dual inhibition of mTORC1 and mTORC2 by the TAK228 has shown significant extension of survival in a murine DIPG orthotropic animal model [60]. However, the therapeutic efficacy of this new inhibitor has not yet been investigated in human DIPG orthotropic animal models.

In conclusion, our findings provide the first *in vitro* evidence that dual inhibition of mitochondrial protein ANT and mTOR represents a novel therapeutic approach for DIPG. The cytotoxic effects appear to be mediated through increased ROS production, depolarized mitochondria and subsequent apoptosis. This combination was found to influence key proteins involved in gliomagenesis such as PDGFRa/PI3K and mTOR. Moreover, combined treatment reduced ATP levels, activated the energy and redox balance sensor AMPK, and inhibited HSP90 function. This combination therapy was found to combine effectively with irradiation. Although *in vivo* efficacy was not demonstrated possibly due to low penetration of the inhibitors into the brain, potential use of more direct delivery methods such as CED, or alternative compounds with better BBB permeability, may ultimately lead to translation of this novel therapy to the clinic.

## METHODS

### Human DIPG neurosphere-forming cultures

Primary DIPG lines were grown as previously reported in stem cell media containing half quantity of DMEM/F12 and half of Neurobasal medium (Invitrogen) supplemented with Glutamax, Pyruvate, Non-essential amino acids, Hepes buffer, antibiotic/antimycotic (Invitrogen), heparin (Stem Cell technologies), human EGF, human basic FGF, PDGF-AA, PDGF-BB (Stem Cell Technologies). Normal Healthy Astrocytes (NHAs) (Lonza) were grown according to the manufacturer’s instructions. All cells were maintained in a humidified atmosphere at 37° C and 5% CO_2_.

### Proliferation assays and combination HTS

DIPG cells were plated at a cell density of 3000 cells/well in 96 well plates and incubated for 72 h. Cells were then treated for 72 h with PENAO, temsirolimus, 17AAG, and combination at the indicated doses. *In vitro* drug sensitivity was assessed with the resazaurin assay (Sigma-Aldrich).

Synergy was evaluated by the method of Chou and Talalay (23) and the data subsequently analyzed by the median effect method using CalcuSyn software (Biosoft). Combination index (CI) values were calculated for each drug combination, where synergy is indicated by a CI less than 1, additive where CI is equal to 1, and antagonism where CI is more than 1.

### Soft agar colony assays

The effects of PENAO, temsirolimus and Irradiation on colony formation ability of the DIPG cells were assessed by soft agar colony formation assay. The assay was performed in 24-well plates. In each well, 300 uL of 0.6% agar (in culture medium) was layered in the bottom followed by 300 ul of 0.3% agar as the top layer. Approximately 3000 HSJD-DIPG007 and SU-DIPG-VI cells were the plated with the top layer and treated with indicated doses. Cells were maintained at 37° C in a humidified 5% CO_2_ atmosphere for 2–3 weeks. The colonies were counted using MTT and presented as percentage colony formation compared to untreated.

### Mitochondrial function and apoptosis flow-cytometric assays

A total of 250,000 DIPG cells were plated and cultured for 72 hours. Subsequently cells were treated with the indicated dose of drugs or irradiation, and incubated for an additional 6, 24 or 48 h. For Apoptosis assays cells were collected for analysis, washed once in cold PBS, and resuspended in 100 uL of Annexin-binding buffer containing 5 ul of Annexin V-FITC and 5 ul of 7AAD (BD Biosciences) according to the manufacturer’s instructions. Subsequently cells were further resuspended with 400 ul of Annexin-binding buffer and fluorescence activated cell sorting (FACS) analysis was conducted on Facs Canto (BD Bioscience), while 10,000 events were collected for each sample.

For mitochondrial function analysis treated cells were stained with 1 uL of MitoSox Red to determine ROS production or 1 uL of JC1 (Invitrogen) to determine membrane polarization according to the manufacturer’s instructions. Stained cells were collected, centrifuged at 1500 rpm for 5 min and resuspended in 500 ul of Phosphate Buffered Saline (PBS) and analysis was conducted as stated above. As positive controls DIPG cells were treated with Anisomycin or CCCP for 2 hours.

### Gene expression analysis

Total RNA was isolated using the Qiagen RNA extraction kit according to the manufacturer’s protocol. First-strand cDNA was synthesized from 1 ug of total RNA using SuperScript III with random primers and oligo(dT)12-14 (Invitrogen). Subsequently 100ng of cDNA was loaded into RT2 Profiler array specific for mTOR pathway. Quantitative PCR reactions were conducted according to the manufacture’s instructions using the Prism 7900 Sequence Detection System (Applied Biosystems). Relative mRNA levels were calculated by the comparative threshold cycle method by using 4 housekeeping genes as controls (19). The results were then expressed as fold changes of cycle threshold (Ct) value relative to controls.

### Western blot analysis

Whole-cell extracts were obtained using RIPA Buffer according to manufacturer protocol, (Sigma-Aldrich) and were quantified by BCA Protein Acid Assay Kit (Pierce). Proteins were resolved on 12–20% Tris-HCl SDS-PAGE precast gels (Bio-Rad) and blotted (Bio-Rad) as described by manufacturer’s instructions. Cell lysates were analyzed with the following antibodies PDGFRa (1:1,000, Cat No:3164), PT849-PDGFRa (1:1,000 Cat No:ab79318) PI3K (110a) (1:1,000, Cat No:4255), PI3K(85) (1:1,000 Cat No:4257) P-PI3K(85) (1:1,000, Cat No:4228), AKT (1:4000, Cat No:4691), PT308-AKT (1:2000, Cat No:9275), PS347-AKT (1:2,000, Cat No:4058), mTOR (1:10,000, Cat No:2983 ), P2448-mTOR (1:2,000, Cat No:5536), p70S6K (1:5,000, Cat No:9202), P-P70S6K (1:2,000, Cat No:9208), 4EBP1 (1:10,000, Cat No:9644), P-4EBP1 (1:2,000, Cat No:9451), Raptor (1:2,000, Cat No:2280), Rictor (1:2,000, Cat No:2114), AMPK (1:1,000, Cat No:5831), P-AMPK (1:1,000, Cat No:2535), HSP90 (1:2,000, Cat No:4875), ANT2 (1:2,000, Cat No:14671), GAPDH (1:5,000, Cat No:97166). All antibodies were obtained from Cell Signaling Technology apart from P-PDGFRa which was purchased from Abcam).

### ATP assay measurements

DIPG cells were plated at a cell density of 10000 cells/well in white opaque 96 well plates and incubated for 24 h. Cells were then treated for 24h with PENAO, temsirolimus, and combination at the indicated doses. ATP levels were assessed using the ATP-Glo assay according to the manufacturer’s instructions (Promega).

### Intracranial DIPG Cell Injection and drug treatments

Pathogen-free, 5 week-old female NOD/SCID mice were purchased from Animal Resources Center (Perth, Australia) and kept at an ambient temperature of 22° C under a 12-hour light cycle (7:00 am**–**7:00 pm). DIPG cells (250,000 cells) were re-suspended in 2 μl of matrigel and injected intracranially into the brainstem of weaned NOD/SCID mice by using a stereotactic device (Kopf Instruments) (coordinates: 0.5 mm anterior, 5.7 mm lateral, 3.5 mm depth from the bregma). Mice exhibiting clinical signs of neurologic decline such as ataxia, circling, head tilting with or without 20% weight loss were humanely euthanased for histological analysis of tumours. Brains were fixed in 10% with formalin neutral buffered solution (Sigma-Aldrich) embedded in paraffin wax and 5-mm sections were cut and mounted on glass slides. Following dehydration sections were stained with hematoxylin/eosin and Ki67 for histologic examination.

PENAO treatments were performed using two different doses and administration routes. PENAO was firstly administered through subcutaneous implantation of the Alzet osmotic minipumps model 2002 (Alzet). The minipumps had a reservoir of approximately 200 ul and were loaded with PENAO dissolved in saline. Continuous administration was performed with animals receiving 3 mg/kg daily at a constant rate of 0.5 ul/hr for 2 weeks. Subsequently minipumps were replaced and treatment continued for another 2 weeks. PENAO was also administered on a second experiment intraperitonealy at 10 mg/kg/day 5days/week for 4 weeks. On the first experiment temsirolimus was dissolved in 0.9%Saline with 5% PEG and 5% Tween80 and subsequently administered intraperitonealy (IP) at 10 mg/kg/day 5 days/week for 2 weeks followed by another 2 weeks at 5 mg/kg/day. On the second experiment temsirolimus was administered intraperitonealy (IP) at 10 mg/kg/day 5 days/week for 4 weeks. Vehicle control mice received pumps containing saline as well as IP administration of vehicle used to dissolve Temsirolimus.

All animal experiments were performed according to the Australian Code of Practice for the Care and Use of Animals for Scientific Purposes under the Animal Research Regulation of the New South Wales (Australia) and under a protocol approved by the Animal Use and Care Committees of University of New South Wales.

### Detection of PENAO in DIPG xenografts

Animals were intracranialy injected with DIPG cells as described above and treated with PENAO through continuous administration with Alzet minipumps (3 mg/kg/day 7 days/week 4 weeks). At the end of the treatment period animals were euthanased and brains were harvested and were immediately snap frozen. Subsequently brainstem and cerebellum regions were lysed with 1ml of 70% w/w nitric acid. Lysates were diluted further with miliq water and analysed for arsenic atoms using an Elan 6100 Inductively Coupled Plasma Spectrometer (Perkin Elmer) [61].

### Statistical analysis

Data were analyzed with GraphPad Prism 5 using an unpaired Student *t* test. All tests are two tailed. *P* values less than 0.05 were considered to be statistically significant. Results are displayed as average ÷ SEM.

## SUPPLEMENTARY MATERIALS FIGURES



## Abbreviations

ADP: Adenosine diphosphate
ACVR1: Activin A receptor type 1
ANT: Adenine nucleotide translocase
AMPK: 5′ adenosine monophosphate- activated protein kinase
ATP: Adenosine triphosphate
BBB: Blood brain barrier
CSF: cerebrospinal fluid
DIPG: Diffuse intrinsic pontine glioma
4EBP1: Eukaryotic translation initiation factor 4E-binding protein 1
EGFR: Epidermal growth factor receptor
ErbB2: avian erythroblastosis oncogene B2 / human epidermal growth factor receptor 2
H3K27M: Histone 3 lysine 27 mutated to methionine
H3.1: Histone 3 isoform 1
H3.3: Histone 3 isoform 3
HSP90: Heat shock protein 90
IP: intraperitoneal
mTOR: Mammalian target of rapamycin
mTORC: mTOR complex
NHA: normal healthy astrocytes
PDGFR: Platelet-derived growth factor receptor
PI3K: Phosphatidylinositol-4,5-bisphosphate 3-kinase
P70S6K: Ribosomal protein S6 kinase beta-1
ShRNA: short hairpin RNA
ROS: Reactive oxygen species
RTK: Receptor tyrosine kinases
TSC1: Tuberous Sclerosis 1
