# Supplementary material for: Dual targeting of mitochondrial function and mTOR pathway as a therapeutic strategy for diffuse intrinsic pontine glioma
Source: Oncotarget. 2018 Jan 8;9(7):7541–56. doi: 10.18632/oncotarget.24045 (PMC5800923; doi:10.18632/oncotarget.24045)
Supplement: Supplementary file 1 [file oncotarget-09-7541-s001.pdf]

Dual targeting of mitochondrial function and mTOR pathway as a therapeutic strategy for diffuse intrinsic pontine glioma

SUPPLEMENTARY MATERIALS

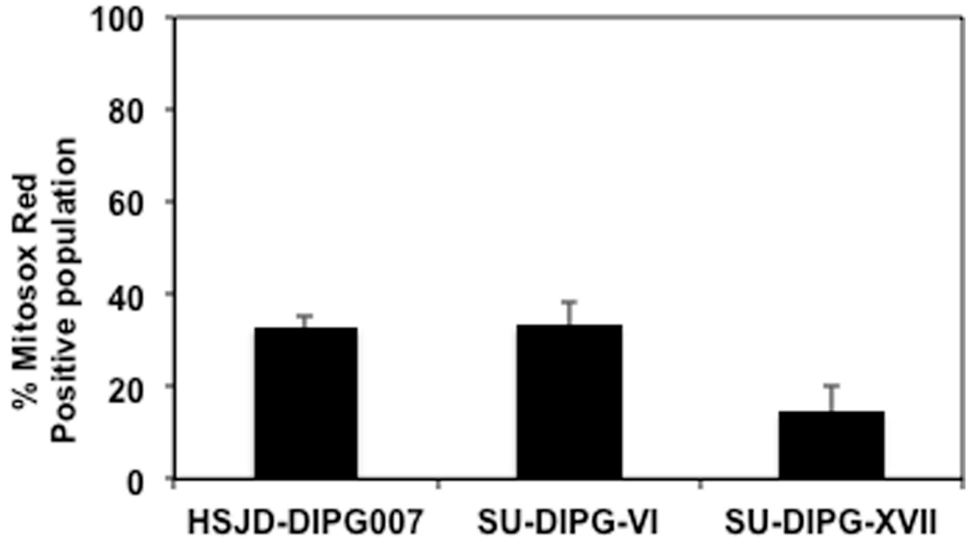

Supplementary Figure 1: Flow cytometric analysis of neurosphere-forming DIPG cells for production of mitochondrial ROS. DIPG cells were stained with MitosoxRed and analysed with FACS Canto B. Data represent average and SD of 3 determinations.

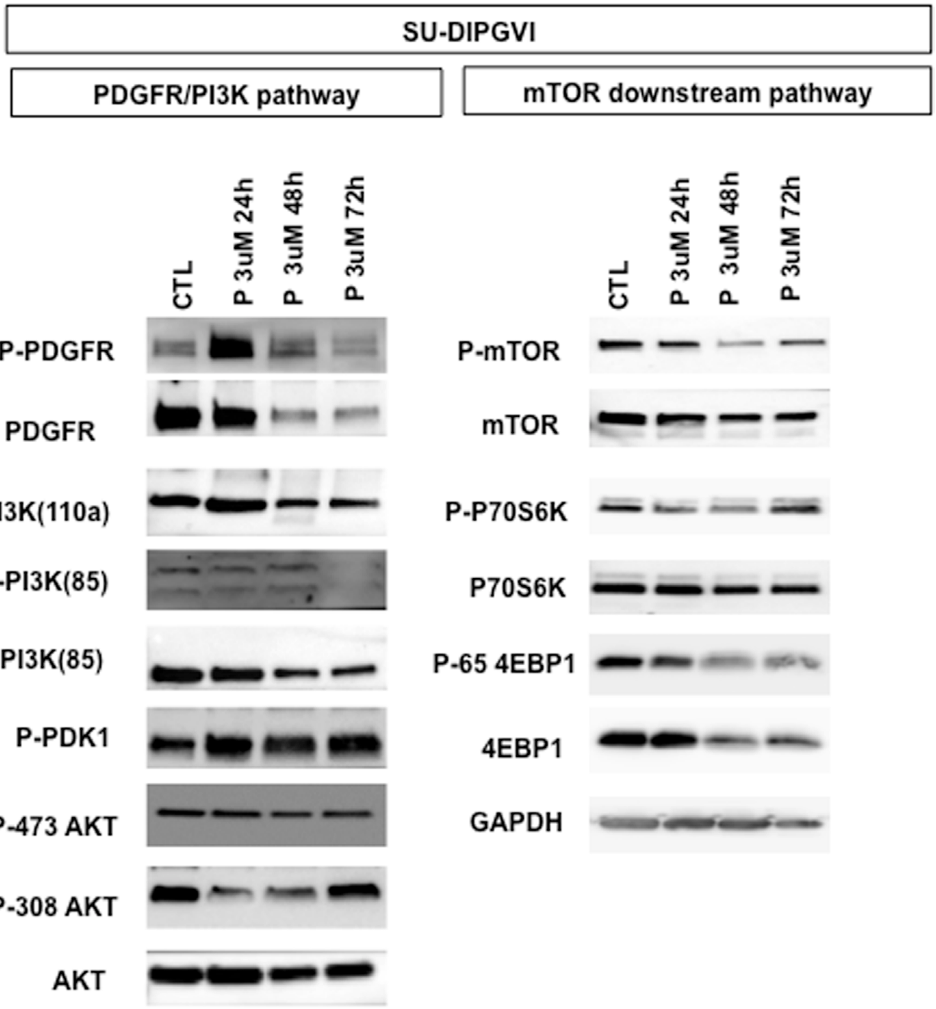

Supplementary Figure 2: PENAO affects PDGFR/PI3K/mTOR pathway in SU-DIPG-VI cells. Representative western blot images of key players involved in PDGFRa/PI3K/mTOR pathway in SU-DIPG-VI neurospheres treated with PENAO as a function of time. DIPG cells were treated with 3 µM of PENAO for 24 h, 48, 72 h and subsequently lysed pellets were examined by western blotting.

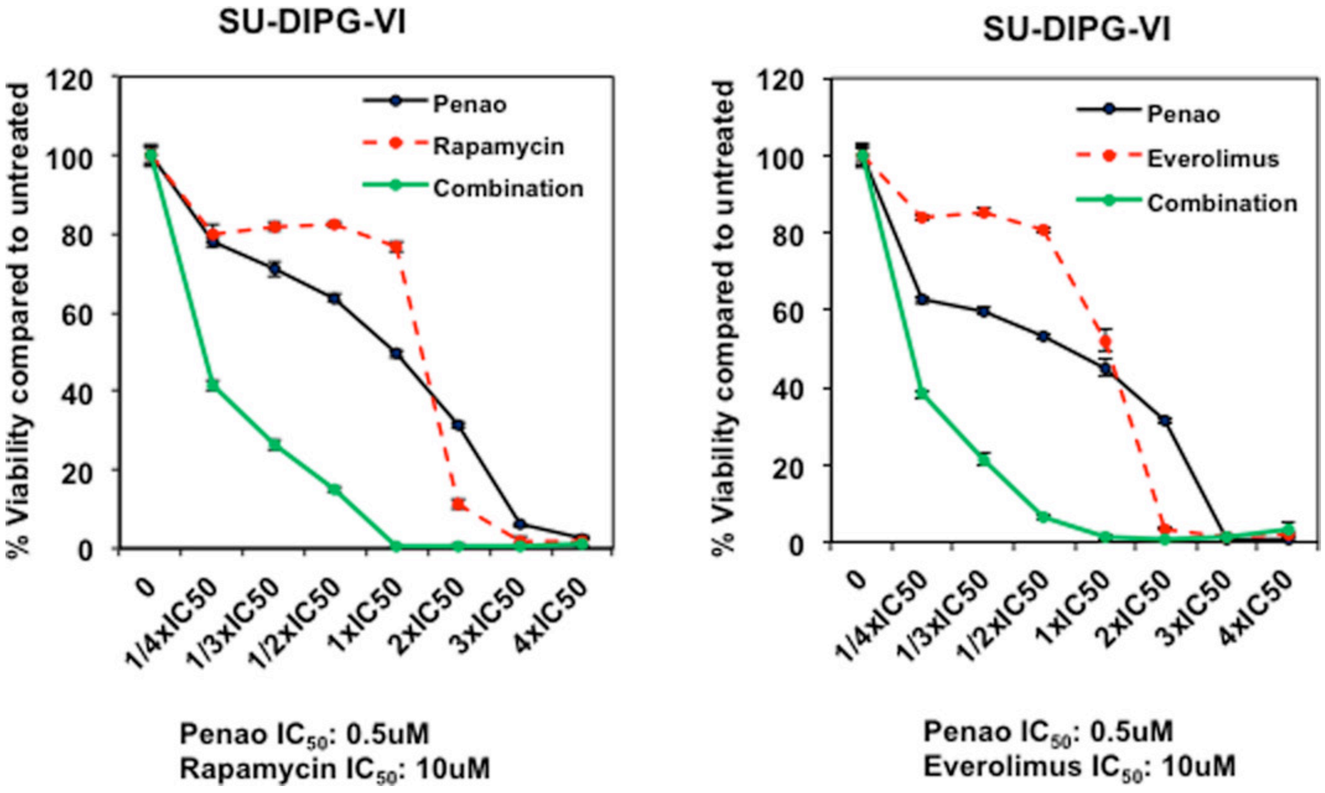

Supplementary Figure 3: Cytotoxic efficacy of PENAO can be enhanced with Rapalogue drugs. Cytotoxic efficacy of PENAO combined with Rapamycin and Everolimus tested at IC_{50} fractions in neurosphere-forming SU-DIPG-VI cells. Viability was assessed by resasurin assay and presented as percentage viability compared to untreated cells. Experiment was replicated 2 times each time $N = 10$.

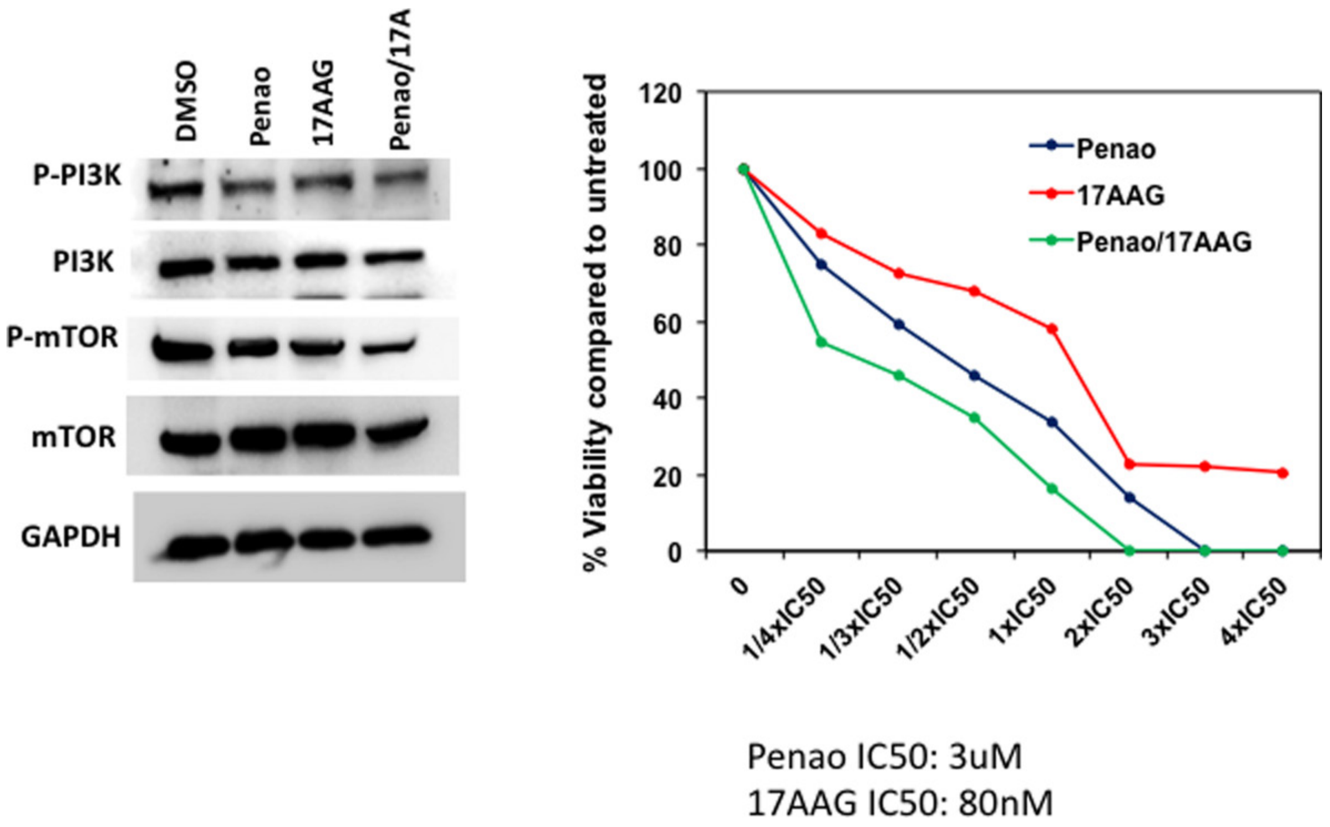

Supplementary Figure 4: Cytotoxic efficacy of PENAO can be enhanced with HSP90 inhibitor 17AAG. Cytotoxic efficacy of PENAO combined with 17AAG tested at IC_{50} fractions in neurosphere-forming HSJD-DIPG007 cells. Viability was assessed by resasurin assay and presented as percentage viability compared to untreated cells. Experiment was replicated 2 times each time *N* = 10. Western blot analysis of PI3K and mTOR upon treatment with PENAO/17AAG combination.
